# Impact of dietary inclusion of *Chenopodium quinoa* on growth performance and survival of Hubbard chicken

**DOI:** 10.1371/journal.pone.0276524

**Published:** 2022-10-20

**Authors:** Samina Mustafa, Muhammad Ahsan Riaz, Muhammad Shareef Masoud, Muhammad Qasim, Ayesha Riaz

**Affiliations:** 1 Department of Zoology, Government College Women University, Faisalabad, Pakistan; 2 Department of Environmental Sciences and Engineering, Government College University, Faisalabad, Pakistan; 3 Department of Bioinformatics and Biotechnology, Government College University, Faisalabad, Pakistan; Sejong University, REPUBLIC OF KOREA

## Abstract

The poultry sector is the most vibrant segment of the agriculture system plays a vital role in the supply of healthy meat products. Broiler production effectiveness is greatly associated with feed formulation. Although, broiler exhibits a relatively fast growth rate, the nutritional profile of its meat has been criticized under conventional human dietary regimes. In the current study, the dietary inclusion of quinoa was assessed to improve broiler growth performance, carcass quality, and health by analyzing different growth, hematological and biochemical, immunological parameters. In the present study, the chicken was fed with 50 g/kg, 100 g/kg, and 200 g/kg quinoa enriched diets in two different experimental groups during the growth phase or finisher phase while chicken fed with diet without quinoa were as control. The 50 g/kg quinoa supplemented chicken group revealed a substantial difference in growth performance in comparison with the control group. In addition, the examination of quinoa dietary supplementation on carcass quality exhibited variable behavior. Further, all the study groups fed with quinoa during the growth phase revealed no remarkable difference in the hematological profile in contrast to the control group except for the chicken group fed (50 g/Kg) during the finisher phase for hemoglobin levels. Likewise, all the quinoa enriched diet given chicken groups showed no significant difference in serum biochemical profile in contrast to the control group except for the 50 g/Kg quinoa fed chicken group during the finisher phase for total globulin levels. In addition, the examination of quinoa dietary supplementation on the broiler serum lipid profile was also assessed and birds exhibited variable behavior as the result of quinoa dietary supplementation. Evaluation of short-term immune response after quinoa supplementation assessed and birds exhibited no marked significance on expression outcomes of interleukin/cytokines (IL 1 beta, IL-6, IL-10) assessed by qRT-PCR analysis. In conclusion, the dietary supplementation of broiler fed with quinoa seeds can enhance the growth performance and the carcass quality of broiler.

## Introduction

Commercial poultry farming with the introduction of hybrid broilers strains started in Pakistan in 1963. Poultry farming, processing recognized by the government as food industry became a sector with a complete investment of 200 billion rupees till 2008 [1]. The private sector’s large investment in the commercial poultry sector in the early seventies rose to a range of 20 to 30% per annum with an annual growth rate between 10 to 15% [2]. With the continuous growth rate in broiler parent stock at the rate of 135%; however, due to the shortage of poultry training facilities in Pakistan, and unqualified people, the poultry industry is still unable to fulfill the protein demand [3]. The higher nutritional protein needs of broilers demand the diet programs with possible effects on the overall growth performance of broilers [4]. Additionally, it demands the most efficient proteins resources that can be applied to chicken diet programs [5].

Nowadays, a popular trend is meat deemed to be "superfood" and "exclusive meat" and this status, among other factors, owes to high contents of antioxidants, proteins, vitamins in addition to macro-and microelements (calcium, iron, phosphorus) in meat [6]. The dietary inclusion of antioxidants can improve broiler performance and immunity [7]. The supplementation of broiler feeds with plant-derived compounds which are rich in antioxidants could enhance oxygen scavenging responses both outside and inside the body [8]. Nutritive stable feeding is of great significance for economic poultry production. Many feed extracts are used to enhance poultry yield including various drugs, but antibiotic-resistant and excessive use of drugs cause a diversity of health risks to consumers [9].

Quinoa (*Chenopodium quinoa*) a wild crop that is cultivated under elevated stress conditions, has high nutritional significance due to increased contents of vitamins (B1, B9, C, and E) and healthy lipid also containing excellent protein amino acid balance [10, 11]. Quinoa seed contains nine essential amino acids with the highest contents of amino acid lysine and methionine, phytochemicals, flavonoids, phenolic acids, squalene, phytosterol, saponins, and multiple other compounds are present in quinoa [12]. The high content of chemical compounds including tocopherols and polyphenols in quinoa seeds can improve blood glucose levels [13–15]. The development of high-quality food products with improved health benefits is gaining attention worldwide. Different varieties of quinoa may provide promising high-quality protein sources [16]. Quinoa having increased- protein grains are a good source of naturally occurring antioxidant compounds makes it imperative as a fodder crop in the poultry and livestock industry [17–19].

Plant extracts enhance the activity of antioxidant enzymes [20] and regulate muscular health and growth [21]. Plant extracts can aid muscle regeneration and enhance anti-inflammatory response [22]. Different studies reported diverse potential health benefits of plant extracts and consumers to prefer to buy meat from broilers fed with plant extracts. Taking into consideration the possible benefits of naturally occurring plant extracts, the present study was designed to evaluate the effects of the dietary inclusion of quinoa seed extracts on the growth performance, carcass characteristics, health, and short-term immune response of broiler chicken [23].

## Materials and methods

### Ethics statement

The study was duly approved by the ethical review committee of Government College Women University Faisalabad-Pakistan. All subjects received humane care according to the guidelines for the care and use of laboratory animals published by National Institute of health.

### Experimental design

The present study was carried out at the Department of Zoology, Government College Women University Faisalabad, Pakistan. A total of 120 one-day-old Hubbard broilers were divided into two major experimental groups and each group is further subdivided into four subgroups/trials with each comprising of five birds and the study was performed in triplicate (Tables 1 and 2). Birds were reared on litter at a stocking density of 0.6ft2/bird for 42 days with a photoperiod of 22 hours of light and 2 hours of darkness. Birds were housed with optimal conditions of temperature, humidity, and ventilation as per breed recommendations.

**Table 1:** Proximate analysis of quinoa.

Constituents	% Dry Weight basis
**Crude Protein**	15.31±0.015
**ASH**	6.21±0.025
**Crude Fat**	3.50±0.038
**Moisture**	8.51±0.032

**Table 2:** Different study groups were used for the examination of quinoa supplemented diet.

**Experimental Group 1**	
Trial 1 (n = 15)	Supplemented with quinoa seeds at 50g/kg (day 15–42) feed
Trial 2 (n = 15)	Supplemented with quinoa seeds, each at 100g/kg (day 15–42) feed
Trial 3 (n = 15)	Supplemented with quinoa seeds, each at 200g/kg (day 15–42) feed
Trial 4 (Control 1) (n = 15)	A diet with no inclusion of quinoa seeds (day 0–42) serving as a control for chicken
**Experimental Group 2**	
Trial 5 (n = 15)	Supplemented with quinoa seeds, each at 50g/kg (day 21–42) feed
Trial 6 (n = 15)	Supplemented with quinoa seeds, each at 100g/kg (day 21–42) feed
Trial 7 (n = 15)	Supplemented with quinoa seeds, each at 200g/kg (day 21–42) feed
Trial 8 (Control 2) (n = 15)	A diet with no inclusion of quinoa seeds (day 0–42) serving as a control for chicken

### Preparation of quinoa supplemented meal

Quinoa seeds were obtained from University of Agriculture, Faisalabad, Pakistan. Freshly harvested seeds were washed with distilled water and dried in the air for three to four days under the shed until attained constant weight for preparation of broiler dietary meal (Table 3). Different composition of quinoa supplemented diets to fed broilers for the proposed study is presented in (Table 2).

**Table 3:** Different compositions of quinoa supplemented diets used to feed broiler chicken.

Ingredients (g/kg)	Control	Trial 1/Trial 3	Trial 2/Trial 4	Trial 3/Trial 6
		50g/ kg	100g/ kg	200g/ kg
Quinoa seeds	-	50	100	200
Wheat	325.0	275.0	220.	110.0
Peas	200.0	202.0	200.	-
Rapeseed (full fat)	150.0	149.9	150.	150
Soybean	184.0	180.0	175.	250
Meat and bone meal (43% crude protein)	60.0	59.0	60.0	60
Molasses	10.0	9.0	10.	-
Soyabean oil	47.5	49.5	46.4	37.5
Calcium carbonate	2.5	3.2	3.2	3.9
Sodium chloride	1.0	1.4	1.2	1.2
DL-Methionine (400gkg**-1)**	6.0	5.9	5.6	3.6
Vitamin	5.0	5.0	4.8	4.8
Crude protein	236	235	227	232
Crude fat	125	127	125	125
Crude fiber	39	44	44	41
ASH	58	57	58	56
Moisture	115	117	117	124

### Growth performance

On day 42 post-birth, all the birds were slaughtered and eviscerated to calculate carcass yield. Carcass yield (%) was calculated using the following equation.


Carcass%=CarcassweightLiveweight×100

### Relative organ weight

After 6 weeks, when birds were 42 days old, both the treated as well as control birds were fasted and weighed on a digital scale (Sartorius model BL 1500). The birds were then bled by puncture of jugular vein to take blood sample. To take blood samples, Jugular vein present on the right side of chicken’s neck was punctured by a sterilized needle. As this vein moves under skin in neck region and blood flows in larger volume, so it is mandatory to immobilize jugular vein to puncture the vein wall. Two persons were required to handle chicken’s body to avoid any injury or pain. One was an operator who held its head in palm of left hand while an assistant restrained the bird’s wings and legs to prevent any body movement. Then, a small quantity of alcohol was applied along with slight pressure at neck base using forefinger. In this way, the vein became prominent and now it was quite easy to get blood sample. Needle was kept inclined (at the angle of 20–25°) to withdraw blood [24]. To obtain different body organs of chicken, they were slaughtered by a humane method for killing. Before their slaughter, they were anaesthetized using an appropriate procedure to lose their consciousness. For this purpose, chicken were placed in gas cabin filled with mixture of 30% O2, 40% CO2 and 30% N2 gases used as anaesthesia [25]. After slaughtering their body organs including gall bladder, spleen, kidney, liver, heart, and lungs were removed and weighed.

### Collection of blood and serum separation

The blood samples were taken into ethylenediamine tetra-acetic acid (EDTA) coated vacutainer for the complete blood analysis and into non-EDTA coated vacationers for blood serum analysis following procedure as reported [24]. Before blood serum collection, samples were incubated for 30 minutes at room temperature then centrifuged at 3000 g for 15 minutes. Finally, blood serum was collected and stored at –20ºC for subsequent analysis.

### Hematological assay

The blood samples collected from broiler were employed for the assessment of various hematological parameters. The determinations of hematological values were carried out at day 42 post-birth after taking 1 ml of blood from the cervical vein of each chicken using 23gauge needles being fixed to a 3 ml syringe. Subsequently, the blood was immediately transferred to a glass tube containing EDTA by gently shaking [25]. Packed cell volume (PCV) levels were measured by using microhematocrit capillary tubes by centrifugation at 2500 rpm for 5 min. Hemoglobin concentration (Hb) was determined employing the Cyanmethemoglobin method as documented [26].

### Serum biochemical profile

On day 42 post-birth, blood serum biochemical profiling was also performed for total albumin and total globulin levels in treated and control birds using a standard commercial kit following the published protocol [27].

### Lipid profiling

The whole blood was collected, incubated, and centrifuged at 4°C for 10 min (1,100 × g). The serum was collected for lipid profile analysis. The levels of Cholesterol, Triglyceride, LDL-C, and high-density lipoprotein cholesterol (HDL-C) were measured following [28] according to the manufacturer’s recommended protocols.

### RNA extraction and cDNA analysis

For RNA extraction liver tissues were collected for gene expression analysis for evaluation Cytokine/interleukin IL-1 beta, IL-6, and IL-10 using TRIzol (Invitrogen, Carlsbad, CA, USA). For cDNA synthesis, five micrograms of total RNA were treated using the StrataScript first-strand synthesis system (Stratagene, La Jolla, CA, USA) according to the manufacturer’s recommendations.

### Real-Time PCR

Quantitative (q)RT-PCR oligonucleotide primers for chicken cytokines and glyceraldehyde 3-phosphate dehydrogenase (GAPDH) internal control is listed in Table 4. Amplification and detection were carried out following the method as described [29].

**Table 4:** The sequence of primers used.

1.	IL 1 beta	Forward	TCGACATCAACCAGAAGTGC
		Reverse	GAGCTTGTAGCCCTTGATGC
2.	IL 6	Forward	AGGACGAGATGTGCAAGAAGT
		Reverse	CAGGTAGGTCTGAAAGGCGAA
3.	IL-10	Forward	GGAGCTGAGGGTGAAGTTTGA
		Reverse	TGATGACTGGTGCTGGTCTG
4.	GAPDH	Forward	GGACACTTCAAGGGCACTGT
		Reverse	TCTCCATGGTGGTGAAGACA

### Statistical analysis

The data were analyzed using the GraphPad Prism 5 software (USA). Results are expressed as mean ± standard deviation (SD). One-way analysis of variance (ANOVA) followed by Dunnett’s post-test was used for analysis. The difference at p ≤ 0.05 is considered statistically significant.

## Results

**Fig 1:**
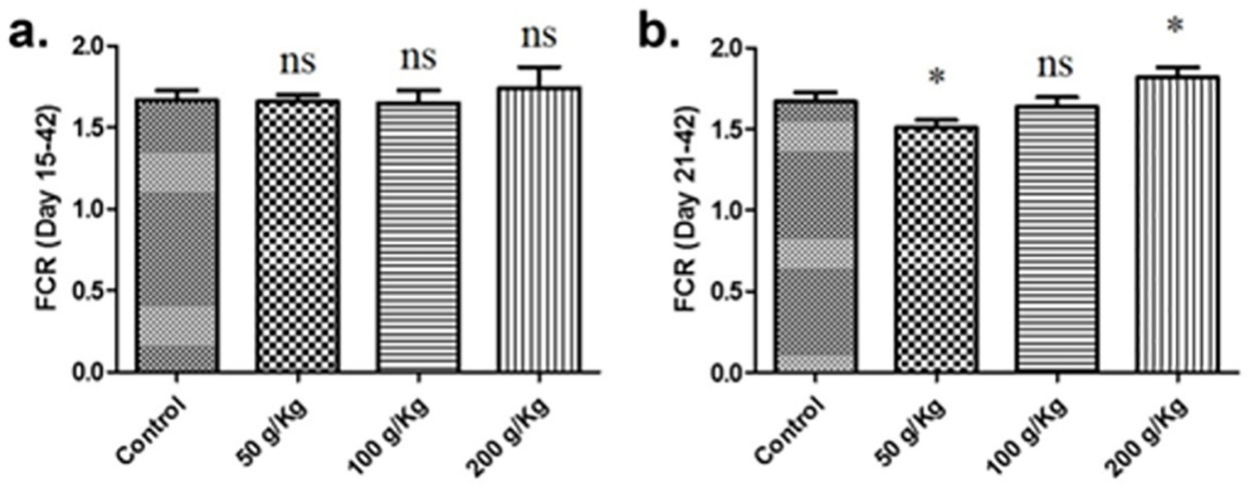
Graphs showing the comparison of feed conversion ratio (FCR) of different quinoa enriched diet given chicken groups. The chickens were given 50 g/Kg, 100 g/Kg and 200 g/Kg of quinoa. (**a**). FCR ratio of chicken fed from days 15 to 42post-birth (**b**). FCR ratio of chicken fed from days 21 to 42 post-birth. All data are expressed as means ± standard deviation (SD) from measurements on 15 chickens. *p≤0.05 in all quinoa supplemented diet fed groups versus the commercially available diet without quinoa fed control group. **Note**: ns means not significant.

In the current study, the impact of quinoa (*Chenopodium quinoa*) on growth performance was studied using Hubbard chicken. Among the study group chicken groups fed with quinoa enriched diets from days 15 to 42 post-birth, no significant difference in the growth performance was observed in comparison with the control group (Fig 1a). In addition, the chicken groups fed with quinoa diets (50g/Kg and 200g/Kg) from days 21 to 42 post-birth exhibited significant difference as compared to the control group while 100 g/Kg quinoa fed chicken group revealed no substantial difference in comparison to the control group (Fig 1b).

**Fig 2:**
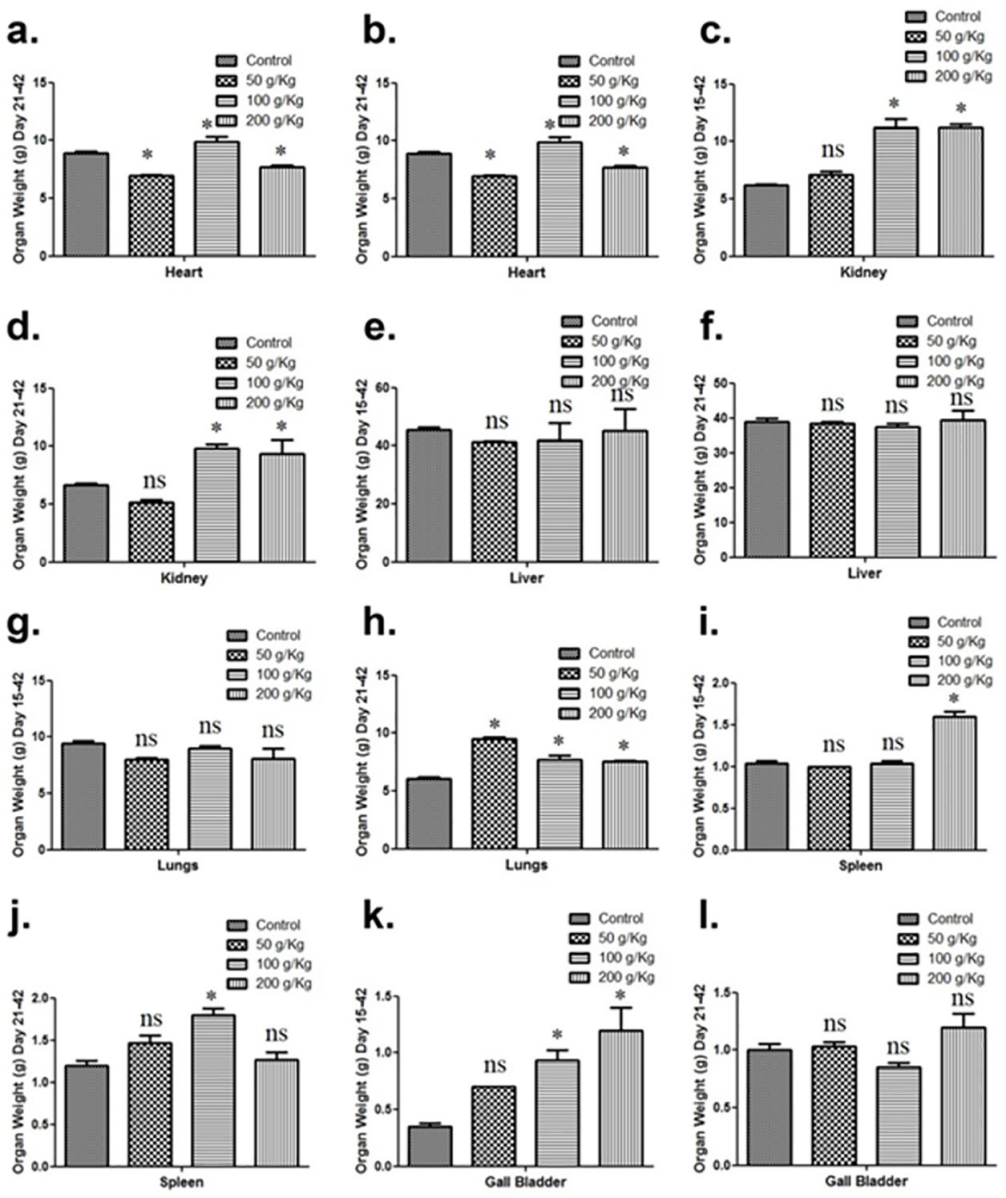
Graphs showing the carcass characteristics of broiler by comparison of various body organ weight of different quinoa enriched diet given chicken groups fed from days 15 to 42 and from days 21 to 42 post-birth. The chickens were given 50 g/Kg, 100 g/Kg and 200 g/Kg of quinoa. (**a**). Heart (Days 15–42) (**b**). Heart (Days 21–42) (**c**). Kidney (Days 15–42) (**d**). Kidney (Days 21–42) (**e**). Liver (Days 15–42) (**f**). Liver (Days 21–42) (**g**). Lungs (Days 15–42) (**h**). Lungs (Days 21–42) (**i**). Spleen (Days 15–42) (**j**). Spleen (Days 21–42) (**k**). Gall bladder (Days 15–42) (**l**). Gall bladder (Days 21–42). All data are expressed as means ± standard deviation (SD) from measurements on 15 chickens. *p≤0.05 in all quinoa supplemented diet fed groups versus the commercially available diet without quinoa fed control group. **Note**: ns means not significant.

The outcomes of quinoa dietary supplementation on the carcass characteristics were observed. Quinoa fed chicken groups (50g/Kg, 100g/Kg) revealed remarkable differences during the growth phase in the weight of the heart in contrast to the control group while in the 200 g/Kg quinoa fed chicken group no significant difference was revealed in the weight of the heart in contrast to the control group (Fig 2a). Moreover, the effects of quinoa dietary supplementation during the finisher phase on the weight of chicken hearts were also evaluated. A significant difference was observed in all the quinoa-fed chicken groups during the finisher phase in comparison with the control group (Fig 2b).

Further, the effects of quinoa dietary supplementation on the chicken kidney were evaluated. All the study groups fed during the growth phase revealed a remarkable difference in the weight of the kidney in contrast to the control group except for 50 g/Kg quinoa fed chicken group revealed no significant difference in the weight of the kidney in comparison with the control group (Fig 2c). Moreover, the impact of quinoa dietary supplementation on the weight of chicken kidneys during the finisher phase was also studied and a significant difference was observed in all the chicken groups in comparison with the control group except for 50 g/Kg quinoa fed chicken group which exhibited no substantial difference as compared to the control (Fig 2d). Next, the impact of quinoa dietary supplementation on the liver was examined during the growth phase and no remarkable difference was revealed in the weight of the liver in contrast to the control group (Fig 2e). Moreover, the effects of quinoa dietary supplementation on the weight of chicken liver were also evaluated during the finisher phase. No significant difference was observed in all the quinoa-fed chicken groups in comparison with the control group in the finisher phase (Fig 2f).

The evaluation of quinoa dietary supplementation on the lungs of chicken revealed that all the quinoa given chicken groups fed during the growth phase revealed no substantial difference in the weight of lungs in contrast to the control group (Fig 2g). Moreover, the effects of quinoa dietary supplementation on the weight of chicken lungs during the finisher phase were also evaluated (Fig 2h) and all the chicken treated with quinoa during the finisher phase exhibited significant differences compared to control.

The impact of quinoa dietary supplementation on the spleen was also evaluated. All the quinoa given chicken groups fed during the growth phase revealed no remarkable difference in the weight of spleen in contrast to the control group except for the 200 g/Kg quinoa supplementation which revealed a substantial difference in comparison with the control group (Fig 2i). Moreover, the effects of quinoa dietary supplementation from days during the finisher phase on the weight of the spleen were also evaluated. The 50 g/kg and 200 g/Kg quinoa fed chicken groups revealed no significant difference during the finisher phase while the 100 g/Kg quinoa fed chicken group exhibited a remarkable difference in comparison with the control group (Fig 2j).

The evaluation of quinoa dietary supplementation on gall bladder of chicken revealed that 100 g/Kg and 200 g/Kg the quinoa given chicken groups fed during growth phase revealed a substantial difference in the weight of gall bladder while 50 g/Kg quinoa fed chicken group revealed no substantial difference in contrast to the control group (Fig 2k). Moreover, the effects of quinoa dietary supplementation on the weight of chicken gall bladder were also evaluated during the finisher phase and exhibited no significant difference in comparison with the control group (Fig 2I).

**Fig 3:**
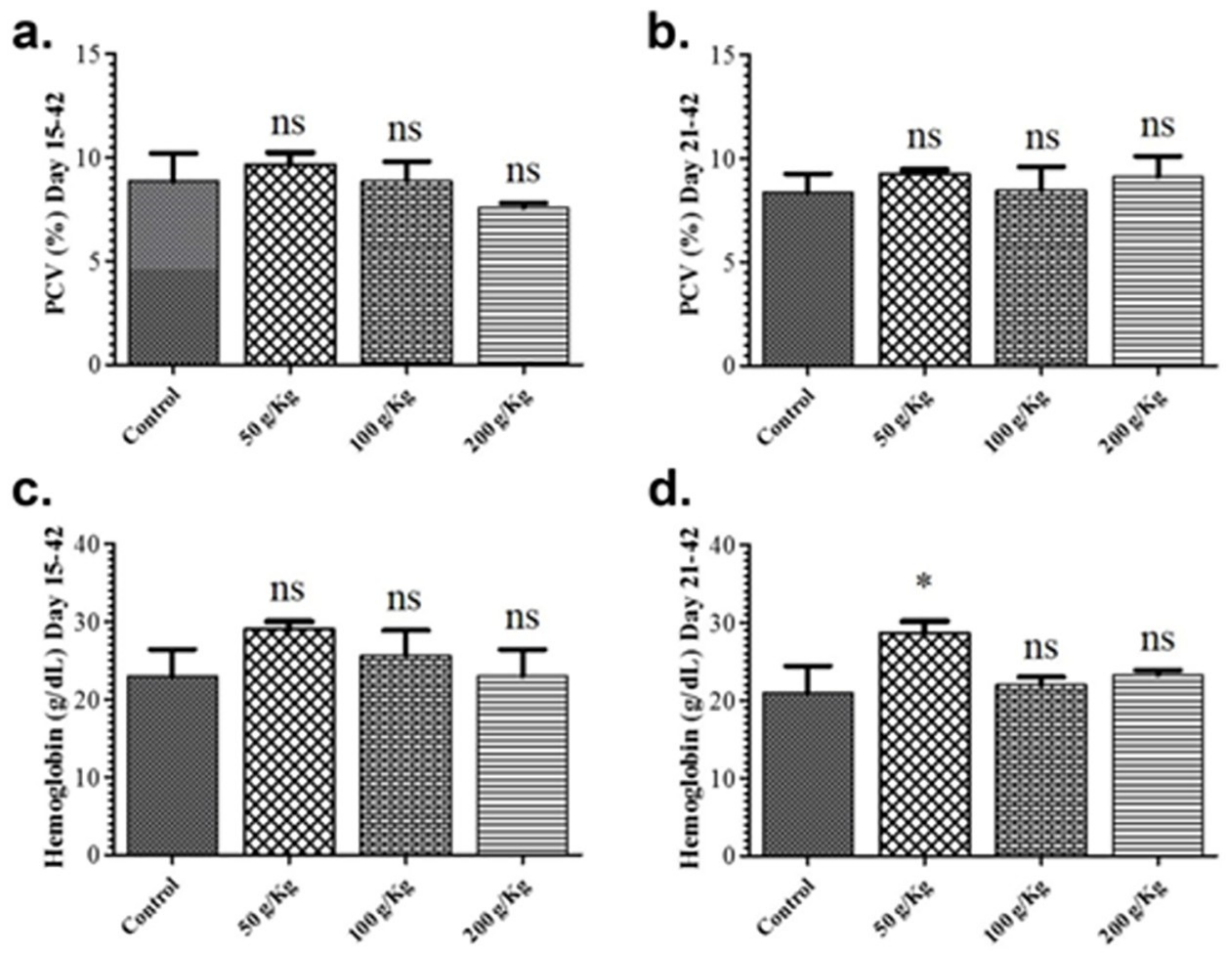
Graphs show the comparison of hematological parameters of different quinoa enriched diet given chicken groups fed from days 15 to 42 as well as from days 21 to 42 post-birth. The chickens were given 50 g/Kg, 100 g/Kg and 200 g/Kg of quinoa. (**a**). Packed cell volume (PCV) levels of chicken treated from days 15–42 (**b**). PCV levels of chicken treated from days 21–42 (**c**). Hemoglobin levels of chicken treated from days 15–42 (**d**). Hemoglobin levels of chicken treated from days 21–42. All data are expressed as means ± standard deviation (SD) from measurements on 15 chickens. *p≤0.05 in all quinoa supplemented diet fed groups versus the commercially available diet without quinoa fed control group. **Note**: ns means not significant.

In the present study, the impact of quinoa supplemented diet on different hematological parameters including PCV and hemoglobin was also studied during the growth phase. All the quinoa enriched diets given chicken groups fed revealed no remarkable difference in the PCV and hemoglobin levels in contrast to the control group during the growth phase (Fig 3a & 3c). Likewise, all the quinoa enriched diets given chicken revealed no remarkable difference in the PCV and hemoglobin levels in contrast to the control group during the finisher phase except for the 50 g/Kg quinoa fed chicken group for hemoglobin levels (Fig 3b & 3d).

**Fig 4:**
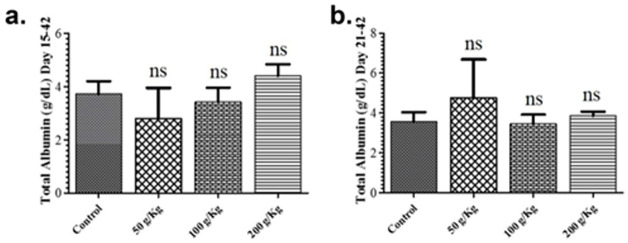
Graphs showing the comparison of total albumin of different quinoa enriched diet given chicken groups. The chickens were given 50 g/Kg, 100 g/Kg and 200 g/Kg of quinoa. (**a**). Total albumin levels of chicken fed from days 15 to 42 post-birth (**b**). Total albumin levels of chicken fed from days 21 to 42 post-birth. All data are expressed as means ± standard deviation (SD) from measurements on 15 chickens. *p≤0.05 in all quinoa supplemented diet fed groups versus the commercially available diet without quinoa fed control group. **Note**: ns means not significant.

**Fig 5:**
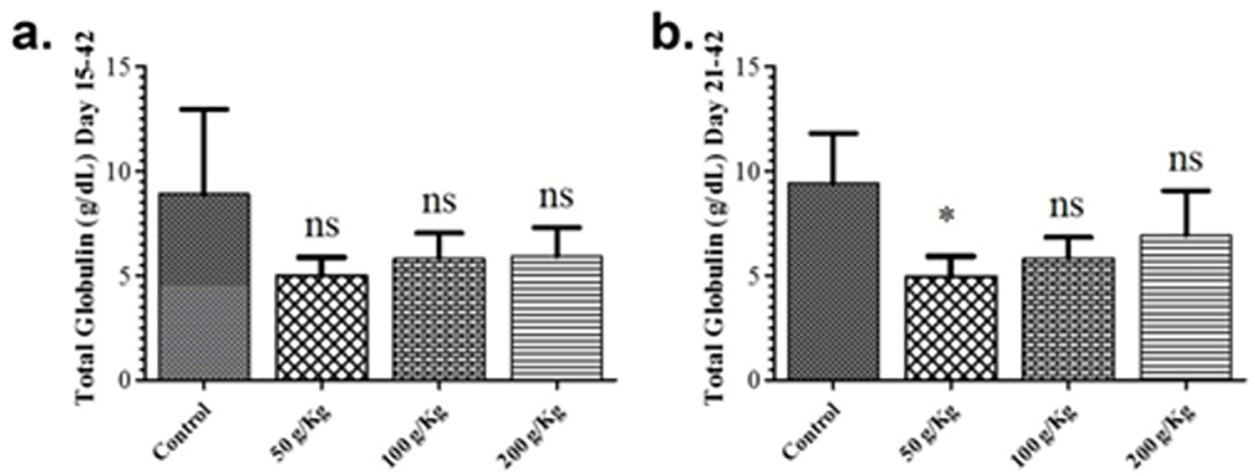
Graph showing the comparison of total globulin of different quinoa enriched diet given chicken groups. The chickens were given 50 g/Kg, 100 g/Kg and 200 g/Kg of quinoa. (**a**). Total globulin levels of chicken fed from days 15 to 42 post-birth (**b**). Total globulin levels of chicken fed from days 21 to 42 post-birth. All data are expressed as means ± standard deviation (SD) from measurements on 15 chickens. *p≤0.05 in all quinoa supplemented diet fed groups versus the commercially available diet without quinoa fed control group. **Note**: ns means not significant.

The impact of quinoa dietary supplementation on total albumin and total globulin was also studied during the growth phase revealed no remarkable difference in the total albumin and total globulin levels in contrast to the control group (Figs 4a & 5a). Likewise, all the quinoa enriched diet given chicken groups fed during the finisher phase revealed no remarkable difference in the total albumin and total globulin levels in contrast to the control group except for the 50 g/Kg quinoa (Figs 4b & 5b).

**Fig 6:**
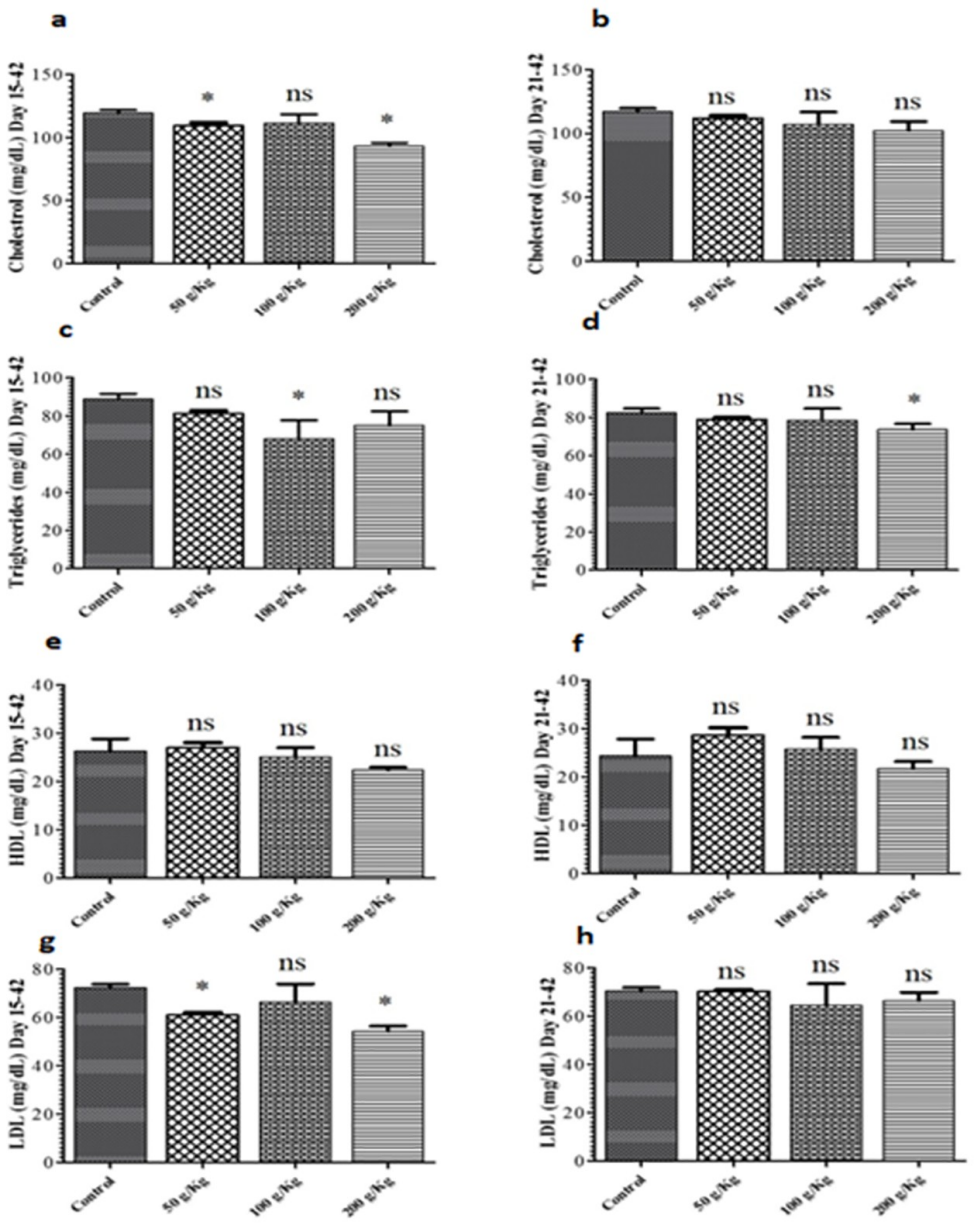
Graph showing the comparison of cholesterol of different quinoa enriched diet given chicken groups fed from days 15 to 42 post-birth (growth phase) and days 21 to 42 post-birth (finisher phase). The chickens were given 50 g/Kg, 100 g/Kg and 200 g/Kg of Quinoa (**a**). Cholesterol level of chicken fed from days 15 to 42 post-birth (**b**). Cholesterol level of chicken fed from days 21 to 42 post-birth (**c**). Triglycerides level of chicken fed from days 15 to 42 post-birth (**d**). Triglycerides level of chicken fed from days 21 to 42 post-birth (**e**). High-density lipoproteins (HDL) of chicken fed from days 15 to 42 post-birth (**f**). High-density lipoproteins (HDL) of chicken fed from days 21 to 42 post-birth (**g**). Low-density lipoproteins (LDL) of chicken fed from days 15 to 42 post-birth (**h**). Low-density lipoproteins (LDL) of chicken fed from days 21 to 42 post-birth. All data are expressed as means ± standard deviation (SD) from measurements on 15 chickens. *p≤0.05 in all Quinoa supplemented diet fed groups versus the commercially available diet without Quinoa fed control group. **Note**: ns means not significant.

The impact of Quinoa dietary supplementation on lipid profile was also assessed. Both 50 g/Kg and 200 g/Kg Quinoa fed chicken groups during the growth phase revealed the remarkable difference in cholesterol levels in comparison with the control group while 100 g/Kg Quinoa fed chicken group revealed no significant difference in cholesterol levels in comparison with the control group (Fig 6a). In contrast, all the Quinoa enriched diet given chicken groups during the finisher phase revealed no remarkable difference in cholesterol levels in contrast to the control group (Fig 6b).

The impact of quinoa dietary supplementation on triglycerides was also studied. Both 50 g/Kg and 100 g/Kg quinoa enriched diet given chicken groups fed during growth phase revealed the remarkable difference in triglycerides levels in contrast to the control group while 200 g/Kg quinoa fed chicken group revealed no significant difference in triglycerides levels in contrast to the control group (Fig 6c). In contrast, both 50 g/Kg and 100 g/Kg quinoa enriched diet given chicken groups fed during finisher phase revealed no substantial difference in triglycerides levels in contrast to the control group while 200 g/Kg quinoa fed chicken group revealed a significant difference in triglycerides levels in contrast to the control group (Fig 6d).

Moreover, the impact of quinoa dietary supplementation on HDL levels was examined. Both 100 g/Kg and 200 g/Kg quinoa enriched diet given chicken groups fed during growth phase revealed no remarkable difference in HDL levels in contrast to the control group while 50 g/Kg quinoa fed chicken group revealed a significant difference in HDL levels in contrast to the control group (Fig 6e). In contrast, no substantial difference in HDL levels was observed in quinoa enriched diet given chicken groups fed during the finisher phase in contrast to the control group (Fig 6f).

Further, the impact of quinoa dietary supplementation on LDL levels was evaluated. Both 50 g/Kg and 200 g/Kg quinoa enriched diet given chicken groups fed during growth period revealed a significant difference in LDL levels in contrast to the control group while 100 g/Kg quinoa fed chicken group revealed no significant difference in LDL levels in contrast to the control group (Fig 6g). In contrast, no substantial difference in LDL levels was observed in quinoa enriched diet given chicken groups fed during the finisher phase in contrast to the control group (Fig 6h).

**Fig 7:**
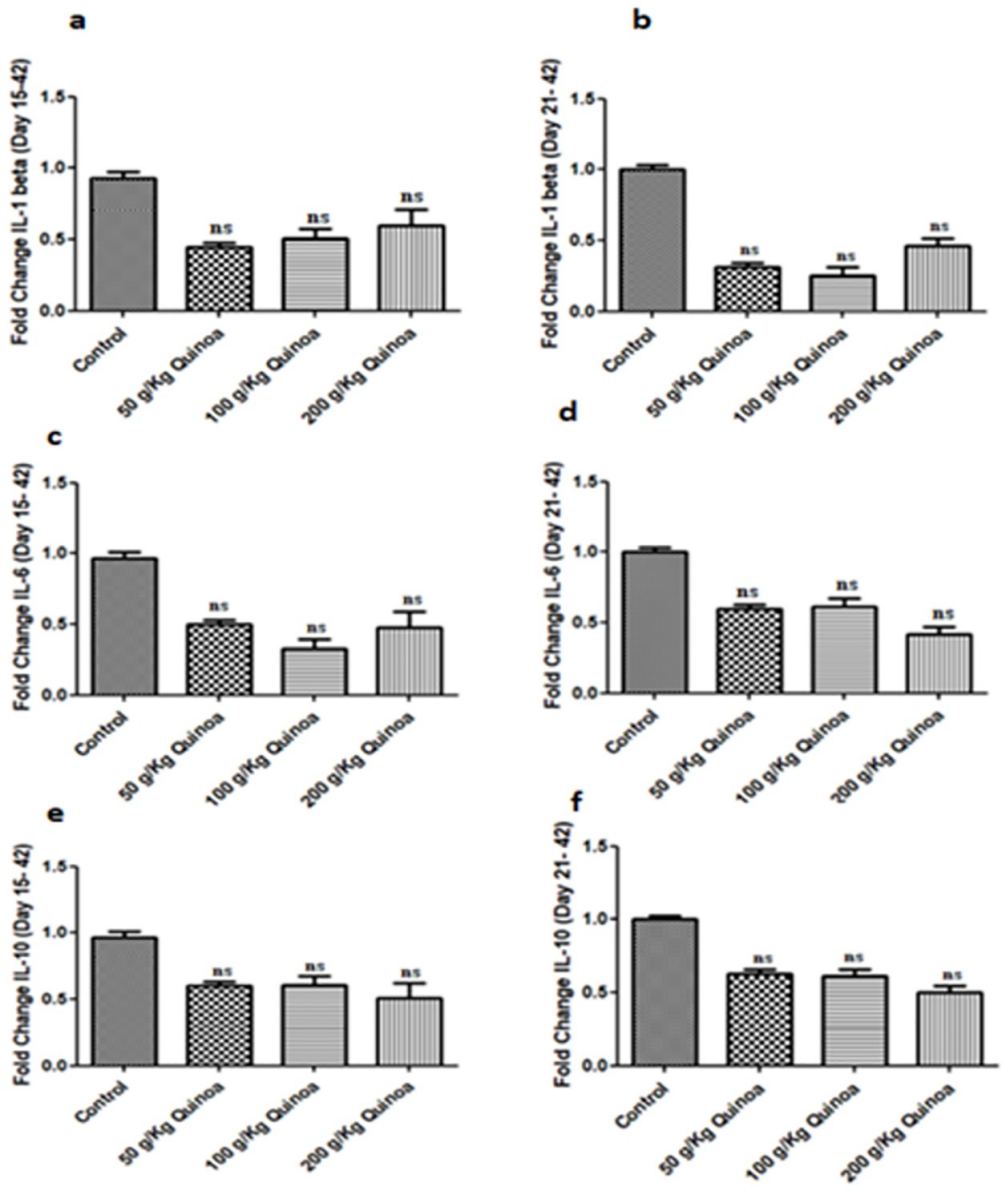
Graph showing the quantitative real time-PCR (qRT-PCR) analysis of liver from different quinoa enriched diet given chicken groups fed from days 15 to 42 post-birth (growth phase) and days 21 to 42 post-birth (finisher phase). (**a**). IL-1 beta (Growth phase) (**b**). IL-1 beta (Finisher phase) (**c**). IL-6(Growth phase) (**d**). IL-6 (Finisher phase) (**e**). IL-10(Growth phase) (**f**). IL-10 (Finisher phase). Expression of IL-1 beta, IL-6, and IL-10 normalized with GAPDH. The chickens were given 50 g/Kg, 100 g/Kg, and 200 g/Kg of quinoa. All data are expressed as means ± standard deviation (SD) from measurements on 15 chickens. *p≤0.05 in all Quinoa supplemented diet fed groups versus the commercially available diet without Quinoa fed control group. **Note**: ns means not significant.

Short-term evaluation of the immune level and nutritional value of dietary inclusion of quinoa on boiler immune makers evaluated for IL-1ß, IL-6, and IL-10gene using Real-Time PCR analysis. To explore any effect of diet supplementation on birds, total RNA isolation and cDNA preparation were made for qRT-PCR analysis. The impact of 50g/Kg, 100g/Kg, and 200g/Kg quinoa supplementation was observed negative for the expression of the IL-1beta gene in the boiler. The qRT-PCR showed non-significant effects among dietary treatments during the growth phase (day 15–42 post-birth) for the expression level of the IL-1 beta gene (Fig 7a). Next, the impact of dietary inclusion of quinoa on the expression of IL-1 beta gene assessed during finisher phase (day 21–42 post-birth) as presented in (Fig 7b). Results showed that the inclusion of quinoa exhibited a non-significant effect on the expression of the IL-1 gene in the broiler.

The impact of dietary inclusion of quinoa on the expression of IL-6 gene assessed during growth or finisher phase as presented in (Fig 7c & 7d). Results showed that the inclusion of quinoa exhibited a non-significant effect on the expression of the IL-6 gene in the broiler in comparison with control. In the current study the impact of dietary inclusion of quinoa on the expression of IL-10 gene in broiler assessed during growth and finisher phase as presented in (Fig 7e & 7f). Results showed that the inclusion of quinoa exhibited a non-significant effect on the expression of the IL-10 gene in the broiler as compared to control.

## Discussion

The current study evaluated the impact of quinoa (*Chenopodium quinoa*) on the growth performance of broiler using Hubbard chicken. The effect of dietary treatments or factors on the broiler growth performance and feed intake were assessed. The effects of dietary inclusion of quinoa seed in feed containing wheat, rapeseed, peas, and soybean meal and were compared with a commercially available feed as the control without quinoa supplementation. In experimental group 1, the broilers received whole quinoa seed feed (50g/kg, 100g/kg, and 200g/kg) during the growth phase to study the effects of quinoa feed additives on average body weight gain, feed consumption, and feed conversion ratio of broiler chicks. Factorial analysis revealed that there was no statistically significant difference in the body weight gain and FCR in contrast to controls during the growth phase. A negligible beneficial effect of inclusion of 100g/kg and 200g/kg quinoa seeds was observed as compared with the control diet during the growth phase. However, the performance of broilers receiving 50 g/kg of a quinoa seed fraction was quite good as compared to control during the growth phase concluding that quinoa in the quantity of 50g/Kg has the potential to serve as broiler feed.

In experimental group 2, the influence of dietary quinoa feed additives on average body weight gain; feed consumption and feed conversion ratio of broiler chicks ’finisher phase was studied using 50g/kg, 100g/kg, and 200g/kg whole quinoa seed meal, compared with a control group without quinoa supplementation. There were statistically significant differences observed in body weight gain and FCR during the finisher phase. The diets supplemented with quinoa feed additives (50g/kg and 200g/kg) significantly improved the body weight gain and FCR of broiler chicks during the finisher period. Findings revealed that the addition of quinoa feed has the potential for tropical feed for poultry, in contrast [30] reported the nutritional value of quinoa, used in large quantities (100–400 g / kg), affected broiler growth performance causing depression in growth rate.

The outcomes of quinoa dietary supplementation on the different body organs were examined. Both 50 g/Kg and 100 g/Kg quinoa fed chicken groups (growth phase) revealed a substantial difference in the weight of the heart in contrast to the control group while 200 g/Kg quinoa fed chicken group revealed no significant difference in the weight of the heart in contrast to the control group. Moreover, the effects of quinoa dietary supplementation during the finisher phase on the weight of chicken heart were also evaluated and a significant difference was observed in all the quinoa-fed chicken groups during the finisher phase in comparison with the control group. All the quinoa enriched diet given chicken groups fed during growth phase evaluated and remarkable difference in the weight of kidney in contrast to the control group was observed except for 50 g/Kg quinoa fed chicken group that revealed no significant difference in the weight of kidney in comparison with the control group. A significant difference was observed in all the quinoa-fed chicken groups during the finisher phase in comparison with the control group except for the 50 g/Kg quinoa-fed chicken group as compared to the controls as no substantial difference was observed.

Further, all the quinoa-fed chicken groups fed during the growth phase or finisher phase revealed no remarkable difference in the weight of the liver in contrast to the control group. The evaluation of quinoa dietary supplementation on the lungs of chicken revealed no substantial difference in the weight of lungs in contrast to the control group during the growth phase while all the chicken (finisher phase) exhibited a significant difference in lungs weight as compared to control.

The impact of quinoa dietary supplementation on the spleen was evaluated and revealed no remarkable difference in the weight of the spleen in contrast to the control group except for the 200 g/Kg quinoa-fed chicken group during the growth phase. Moreover, the 50 g/kg and 200 g/Kg quinoa fed chicken groups revealed non-significant differences while the 100 g/Kg quinoa fed chicken group exhibited a remarkable difference in comparison with the control group during the finisher phase.

The evaluation of dietary treatment on gall bladder of chicken revealed that 100 g/Kg and 200 g/Kg the quinoa given chicken groups revealed a substantial difference in the weight of gall bladder during growth phase while 50 g/Kg quinoa fed chicken group revealed no substantial difference in contrast to the control group. Moreover, the effects of quinoa dietary supplementation during the finisher phase on the weight of chicken gall bladder were also evaluated and exhibited no significant difference in comparison with the control group during the finisher phase.

Dietary inclusion of quinoa (growth phase) significantly affected the proportional weight of gall bladder; kidney and spleen compared to control chickens were recorded. However, a non-significant change in the proportional weights in the lung was recorded during the growth phase. On a cumulative basis, a better relative organ weight was observed after quinoa dietary inclusion as compared to control. Trial 6 with 200g/Kg quinoa was significantly superior to all treatments during the finisher phase while 100g/Kg quinoa dietary treatment (Trial 2) factorial approach revealed significantly (p<0.05) high values during the growth phase.

In the present study, the impact of quinoa dietary supplementation on different hematological parameters including PCV and hemoglobin was also studied. All the quinoa enriched diet given chicken groups fed during growth or finisher phase revealed no remarkable difference in the PCV and hemoglobin levels in contrast to the control group except for the 50 g/Kg quinoa fed chicken during finisher group for hemoglobin levels.

The impact of quinoa dietary supplementation on total albumin and total globulin was also studied. All the quinoa enriched diet given chicken groups fed during growth phase or finisher phase revealed no remarkable difference in the total albumin and total globulin levels except the inclusion of 50g/kg quinoa during the growth phase of broiler significantly improved the serum globulin level in contrast to the control group. Similar to our findings, previous studies also reported variable effects on boiler carcass characteristics perhaps better explaining diversification of energy towards protein accretion in presence of balanced quinoa supplementation in broiler diet [31].

The impact of Quinoa dietary supplementation on broiler lipid profile was also studied. Studies reported the changes in total cholesterol and Triglycerides after the inclusion of quinoa seeds in the diet is in line with [32]. Studies also reported potential inhibitory effects of quinoa on the hepatic synthesis of cholesterol resulted in reduced production of plasma cholesterol [33, 34]. Here, both 50 g/Kg and 200 g/Kg Quinoa dietary inclusion during the growth phase revealed the remarkable difference in cholesterol levels in contrast to the control group in contrast to Quinoa dietary inclusion during the finisher phase compared with the control group.

The impact of Quinoa dietary supplementation on triglycerides was studied. Both 50 g/Kg and 100 g/Kg Quinoa supplementation during the growth phase revealed remarkable differences in triglycerides levels in comparison with the control group were in line with favorable changes in Triglyceride levels [35]. Moreover, the impact of Quinoa dietary supplementation on HDL levels was observed. Both 100 g/Kg and 200 g/Kg Quinoa supplementation during growth or finisher phase revealed no remarkable difference in HDL levels in contrast to the control group while 50 g/Kg Quinoa fed chicken group revealed a significant difference in HDL levels in comparison with the control group. Further, the impact of Quinoa dietary supplementation on LDL levels during the growth or finisher phase was evaluated and revealed that 50 g/Kg and 200 g/Kg Quinoa enriched diet given chicken groups fed during the growth phase significantly affected LDL levels in contrast to the control group is in consistence with [36].

Cytokines, essential markers of immunity are recognized as endogenous signaling molecules that mediate the cellular defense system [37]. Evaluation of short-term immune expression is an important intervention to elevate the general immunity of animals. Nutrition improves the innate immune system however in the current study quinoa dietary supplementation exhibited no remarkable effects on expression outcomes of interleukin IL-1beta was measured. In particular, IL-1β is involved in the inflammatory response and the secretion of the antibody under hyperthermia in animals [38, 39]. In our results where birds fed the diet supplemented with quinoa showed a non-significant effect on hepatic Interleukin 6 (IL-6), a cytokine that is a major mediator of the host response to stress [40]. Similarly, the expression IL-10 was observed non-significant in a study group in contrast to the control group. However, some studies reported that dietary supplementation with antioxidants/vitamins may affect the expression of pro-inflammatory cytokines in rodents [41].

## Conclusion

In conclusion, nutrition has a major influence on the general health of broilers. Due to adverse potential health benefits, broiler meat with natural extracts and inclusion of dietary antioxidant supplements from quinoa plant sources can improve overall the growth performance, carcass characteristics, hematological and biochemical parameters of broiler by minimizing oxidative stress in birds and enhancing the activities of enzymes, ultimately improving the general health of broiler.
